# Real world evidence (RWE) – a disruptive innovation or the quiet evolution of medical evidence generation?

**DOI:** 10.12688/f1000research.13585.2

**Published:** 2018-08-29

**Authors:** Sajan Khosla, Robert White, Jesús Medina, Mario Ouwens, Cathy Emmas, Tim Koder, Gary Male, Sandra Leonard

**Affiliations:** 1 AstraZeneca Academy House, Cambridge, CB2 1PG, UK; 2 AstraZeneca, Gaithersburg, MD, 20878, USA; 3 AstraZeneca, Madrid, 28033, Spain; 4 AstraZeneca AB R&D, Mölndal, 431 50, Sweden; 5 AstraZeneca, Luton, LU1 3LU, UK; 6 Oxford PharmaGenesis, Tubney Warren Barn, Tubney, Abingdon, OX13 5QJ, UK

**Keywords:** Real world evidence, Drug Discovery methods, Drug Industry methods

## Abstract

Stakeholders in healthcare are increasingly turning to real world evidence (RWE) to inform their decisions, alongside evidence from randomized controlled trials. RWE is generated by analysing data gathered from routine clinical practice, and can be used across the product lifecycle, providing insights into areas including disease epidemiology, treatment effectiveness and safety, and health economic value and impact. Recently, the US Food and Drug Administration and the European Medicines Agency have stated their ambition for greater use of RWE to support applications for new indications, and are now consulting with their stakeholders to formalize standards and expected methods for generating RWE.

Pharmaceutical companies are responding to the increasing demands for RWE by developing standards and processes for each stage of the evidence generation pathway. Some conventions are already in place for assuring quality, whereas other processes are specific to the research question and data sources available. As evidence generation increasingly becomes a core role of medical affairs divisions in large pharmaceutical companies, standards of rigour will continue to evolve and improve. Senior pharmaceutical leaders can drive this change by making RWE a core element of their corporate strategy, providing top-level direction on how their respective companies should approach RWE for maximum quality.

Here, we describe the current and future areas of RWE application within the pharmaceutical industry, necessary access to data to generate RWE, and the challenges in communicating RWE. Supporting and building on viewpoints from industry and publicly funded research, our perspective is that at each stage of RWE generation, quality will be critical to the impact that RWE has on healthcare decision-makers; not only where RWE is an established and evolving tool, but also in new areas that have the potential to disrupt and to improve drug development pathways.

## Introduction

In March 2016, the US Food and Drug Administration (FDA) released a statement outlining the goals and procedures for the Prescription Drug User Fee Act (PDUFA) VI for 2018–2022, with notice that this would include the use of real world evidence (RWE) in regulatory decision-making. In December 2016, the 21st Century Cures Act became law in the USA, aiming to expedite approval for new medicines. Towards that aim, it included provision for RWE to be used in place of evidence from randomized controlled trials (RCTs), if judged appropriate by the FDA.

RWE is derived from the analysis of data collected from a healthcare setting, outside the context of prescriptive RCTs ^1^. One of the key objectives of RWE is to understand observations and events in patients in routine clinical practice. RWE complements RCTs, which are carefully controlled experiments to test specific hypotheses on the efficacy and safety of new drugs, and which by design do not reflect current clinical practice. Owing to the mechanism of data collection and experimental design, RWE studies generally may not yield definitive causal inference because of the many confounders of variability ^2, 3^.

The FDA aims to publish draft guidance for the use of RWE by October 2021, and consultation is already underway with healthcare sector stakeholders including the pharmaceutical industry. Both the FDA and the European Medicines Agency (EMA) have stated their wish to see increased use of RWE in supporting indications. In Asia, the growing maturity of real world data sources has led to the recent use of RWE in regulatory discussions, for example, in the decision in Japan on the use of raloxifene for the treatment of osteoperosis ^4^. Indian regulatory authorities are also looking to embed routinely collected electronic health records into their decision-making process ^5^.

To date, use of RWE by the pharmaceutical industry has primarily focused on the peri-launch period just before, and immediately after, marketing approval of a drug, to describe patient populations, to contribute towards knowledge of patient safety and to make judgements on comparative effectiveness between drugs. RWE is also regularly used in economic modelling and when establishing appropriate pricing for new therapeutic interventions. While details of the methods used vary between agencies, RWE is central to the healthcare technology assessments (HTA) by which payers judge if a new drug is cost-effective in their healthcare system ^6^.

Earlier stages of the clinical drug development pipeline are now starting to use RWE to support critical decisions. Experts in RWE from several large pharmaceutical companies have previously published their views on this topic, as have companies that specialize in generating RWE for the pharmaceutical industry, and an overview of results from an unstructured literature search is provided in Table 1 ^7– 13^. Pharmaceutical companies have recognized the demand for RWE from national regulators and other healthcare decision-makers; however, few to date have gained committed support at board level for RWE generation being a critical part of the business. With the focus on specialty care, progress in technology and increasing availability of real world data, the time is right to provide this support to ensure that patients can access the medicines they need.

**Table 1.** Selected industry perspectives on RWE.

Reference	Year	Company	Summary
**Epstein *et al.*^7^**	2012	Sanofi	A cross-functional approach to evidence generation in the pharmaceutical industry, with a real world perspective, will streamline regulatory approval
**Hughes and** **Kessler ^8^**	2014	IMS Health	1. RWE capabilities should converge in a platform 2. Narrow focus on specific therapeutic areas and markets leads to differential value from RWE 3. The commercial organization must champion efforts to broaden RWE’s application and value 4. Speed is a goal
**Berger *et al.*** ^9^	2015	Pfizer	The potential for success of big data to improve healthcare depends on the development of suitable policies, especially regarding openness and quality
**Ronicke, Ruhl** **and Solbach ^10^**	2015	Strategy&, a division of PwC	Companies need to develop specific strategies for RWE, either driven by senior leaders top-down or encouraged to grow in local companies bottom-up
**GSK U.S. Public** **Policy ^11^**	2015	GSK	Proactive communication of RWE will improve healthcare but regulatory guidance is required to define robust research standards
**Galson and** **Simon ^12^**	2016	Amgen and Group Health Research Institute	RWE generation must be driven by the needs of healthcare decision-makers. Integration of clinical research with clinical practice must be facilitated to improve healthcare
**QuintilesIMS** ^13^	2017	QuintilesIMS	Big data, powerful analytics and a strategic approach to collaborations will generate the best insights with greatest transparency
**Andersson** **and Kyhlstedt ^15^**	2017	Synergus	A structured search to identify the relevant RWD sources is an essential step for successful execution of RWE studies

GSK, GlaxoSmithKline; PwC, PricewaterhouseCoopers; RWD, real world data; RWE, real world evidence.

**Figure 1.**
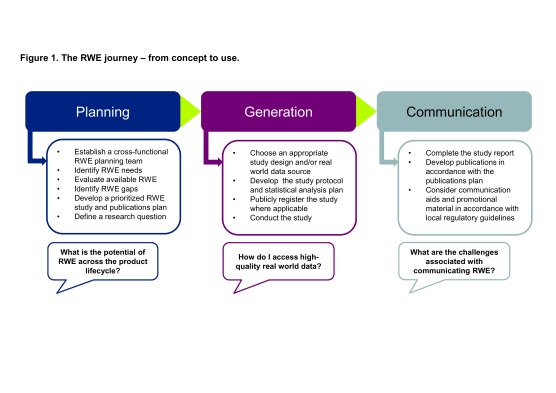
The RWE journey – from concept to use. An outline of the main steps and some key questions in the planning, generation and communication of RWE. RWE, real world evidence.

This perspectives paper is aimed primarily at an industry audience with an interest in forming or expanding an RWE function, and looks to describe the planning, generation and communication of RWE ( Figure 1), specifically:

• the current and future areas of RWE application in the pharmaceutical industry
• the source, quality of and access to data that are necessary to generate RWE
• challenges in communicating RWE.

## Potential for RWE in drug lifecycles

The pharmaceutical industry faces fresh challenges in finding ways to make its innovative medicines available to patients. The interests of healthcare system payers and regulators, and the need to measure disease burden, create a complex environment for quantifying clinical value.

**Figure 2.**
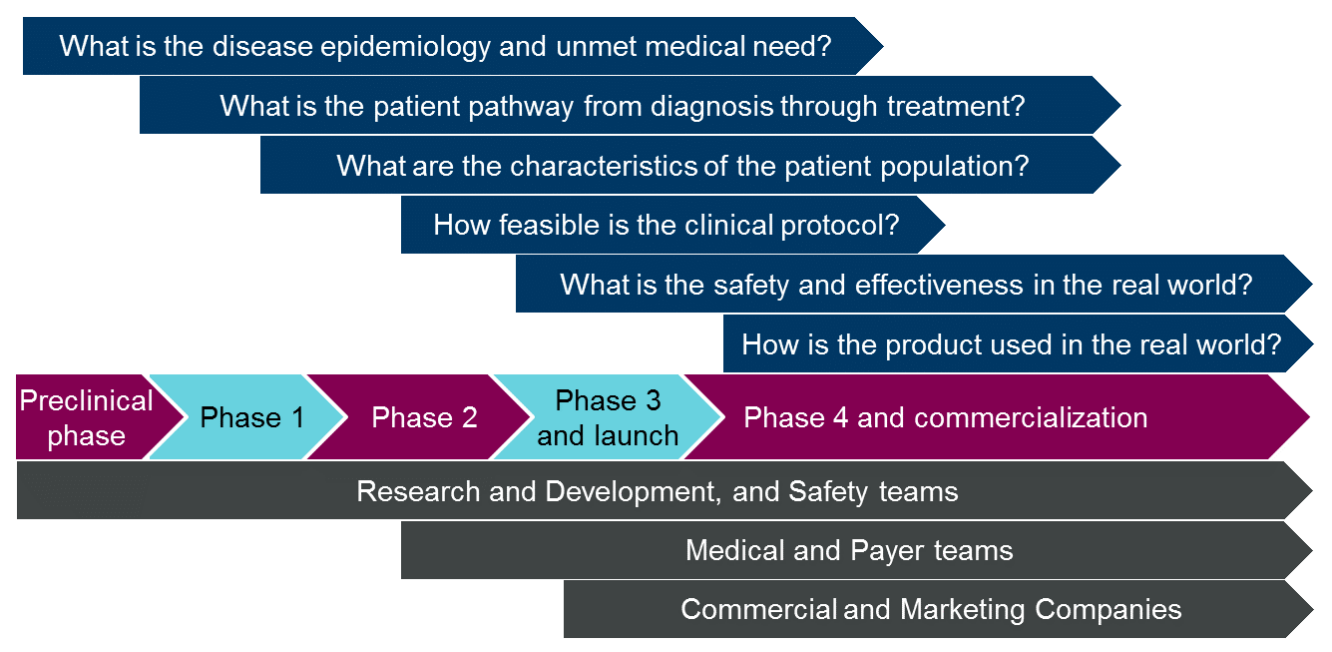
RWE is used throughout the product lifecycle. The questions that can be addressed by RWE and the functions involved in the generation or use of RWE at each stage of product development. RWE, real world evidence.

Continual observation of disease epidemiology, treatment patterns and outcomes in the real world can help to prioritize and to streamline medicine development, with the potential for accelerating evidence generation to support label expansion for specific products. All phases of medicine development can benefit from increased observation of the real world ( Figure 2 and Table 2).

**Table 2.** Evidence needs across the product lifecycle.

Purpose	Evidence needed
**Shape target product profile**	• Understanding unmet needs and outcomes that matter to patients • Describing the potential market, which with pricing decisions leads to forecasts of future earnings and information that affects development investment
**Shape design of phase 3 studies**	• RWE on the patient population, unmet need and standard of care • Evidence on pharmacology, toxicology and efficacy
**Achieve registration/approval**	• Preclinical, toxicology and pharmaceutical chemistry data • Robust efficacy and safety data • Early clinical studies
**Achieve access and reimbursement**	• RWE on the patient population, unmet need and burden of disease • RWE on comparative effectiveness and costs of comparator or standard of care • Cost-effectiveness and budget impact models
**Maintain access and demonstrate** **continued value**	• RWE on comparative clinical effectiveness and costs • Long-term outcomes data • Patient safety data • Patient adherence and utilization data

RWE, real world evidence.

### Disease strategy and early discovery disease segmentation

RWE has the potential to be used early in drug discovery and development programmes, facilitating product development by identifying diseases or indications that represent a significant burden in populations. Electronic health records to support differentiation of patients’ needs have been used within the National Institutes of Health (NIH), and the ability to characterize patient populations before conducting a trial has enabled the NIH to design trials that accelerate innovative interventions to testing phase in patient subgroups of particular need ^14^.

### Phase 1–3 clinical study design

To ensure a clinical trial protocol has internal validity, trial design teams will often use a set of restrictive eligibility criteria that may remove from the trial large segments of a population with the disease of interest. The impact of these eligibility criteria is often not understood or in most cases is not tested until the question of generalizability is raised at the stage of regulatory or reimbursement submission ^16^. This has been recognized as a limitation of RCTs by many regulators, including the FDA, in response to many approved medicines being withdrawn owing to safety problems being identified once a therapy has been exposed to a broad patient population ^17^. RWE can aid clinical study design; for example, in assessing the population size of patients with different sets of inclusion or exclusion criteria.

### Indication-seeking and label-expanding studies

In order to license a therapy in a new indication or to expand the label into a new population, it is mandatory to establish evidence to support the efficacy claim. Traditionally, explanatory trials determine whether the intervention produces the expected result under controlled circumstances, generated through careful design of RCTs. As the need for larger RCTs increases, owing to low-rate event endpoints, potentially differential efficacy throughout subpopulations of patients and the need to observe larger populations for rare adverse events after intervention, the cost of running the trials increases. The time to run these trials also impacts on the potential profitability of indication expansion. Therefore, new thinking is required on how and if explanatory trials can leverage some of the features of real world trials to deliver accelerated efficacy studies.

### Pragmatic clinical trials – a hybrid approach

The main features of an RCT are the randomization of patients, enrolment into a controlled trial setting and follow-up specified in a study protocol. Applying this concept while also using real world data may provide a hybrid approach to running pragmatic clinical trials. The levels of pragmatism can be understood within the context of the PRagmatic Explanatory Continuum Indicator Summary (PRECIS)-2 framework ^18^. In the regulatory context, a balanced approach of using real world data to execute large-cohort phase 3 trials may generate enough of a reward to risk taking the step towards an innovative execution model. This hybrid approach to running studies has been taken in examples such as the Salford Lung Study (see Box 1) ^19, 20^.

Box 1. **Case study: The Salford Lung Study**

The Salford Lung Study assessed the effectiveness and safety of fluticasone furoate in patients with chronic obstructive pulmonary disease (COPD). In this 12 month, open-label, phase 3, multicentre study, 2799 patients with COPD were randomized 1:1 to a once-daily inhaled combination of fluticasone furoate 100 μg and vilanterol 25 μg, or to continuation of their existing therapy. This collaborative study collected data using electronic health records of consenting patients across all of their interactions with general practitioners (GPs), pharmacists and hospitals. In total, 75 GP practices, 128 community pharmacies in Salford and South Manchester, and two hospitals participated in the study ^19, 20^. The primary objective of the Salford Lung Study pragmatic approach was to assess the effectiveness of the treatment, and pragmatic features were not primarily used to decrease costs or to increase speed of delivery. Although the cost of the Salford Lung Study has not been published, the expenditure incurred by the training of healthcare professionals and the development of a bespoke data collection system is likely to be high. The cost of such an approach should therefore be carefully evaluated before it is used to implement such a study.

### Post-authorization studies

Regulators including the FDA, EMA and China Food and Drug Administration increasingly ask pharmaceutical companies to implement ‘post-marketing commitment’ studies as a condition of approval. In some cases, these commitments are requested after a product launch, for example, in light of new safety concerns. The studies may cover safety, efficacy, effectiveness or optimal use. One specific type of study, a post-authorization safety study, is usual for product authorizations: a large group of patients receiving the new medicine is tracked, often for a longer time period than covered by the registrational trial. Pharmaceutical companies are also obliged to enforce systems for spontaneous safety reporting, capturing and assessing adverse event data received from prescribing physicians. These data are consolidated into reports for regulators, and are typically used for pharmacovigilance rather than for public reimbursement by each country’s national and local bodies, based on its effectiveness and safety, value for money and affordability. These are the key questions covered by health technology assessments, answered by health economic models that use data from RCTs and RWE studies, plus financial estimates and calculations ^6^.

Physicians also need to know how best to use new treatments in the broad patient population, not just in the restricted clinical trial sample. To give prescribers, guideline committees and formularies confidence to offer the medicines to patients, companies and independent investigators run retrospective and prospective RWE studies, showing outcomes from treatments in their region ^21^.

## Real world data

Data collected in a routine healthcare setting must be stringently curated, validated and standardized to enable the generation of robust RWE ^1^. Primary real world data are generated specifically for the purposes of the research, through prospective collection from diagnostic or monitoring procedures. Secondary real world studies use data that were routinely collected for medical or administrative purposes – such as electronic health (or medical) records and administrative claims databases – for the generation of RWE. More recently, a complementary source of real world data, generated directly by patients, has emerged from the growth of health-focused online communities and research networks. Sources such as the PatientsLikeMe research network, in which patients are encouraged to share health data in a structured, standardized format, give more scope for formal research. While structured patient-generated data sources such as PatientsLikeMe can lead to useful evidence (see Box 2), researchers should be aware of the limitations of data generated outside the healthcare environment, such as the challenge of how to validate the data. Examples of available real world data sources are provided in Table 3, and a detailed overview of their benefits and limitations is provided in the GetReal RWE Navigator.

Box 2. **Case study: PatientsLikeMe study in amyotrophic lateral sclerosis ^22^**

PatientsLikeMe has an established and engaged community of patients with amyotrophic lateral sclerosis (ALS), a rapidly progressive and fatal neurodegenerative condition with no effective treatments. Approximately 9% (348) of patients with ALS in the PatientsLikeMe community reported using lithium carbonate, a drug which had shown promise in a small study (16 treated patients, 28 controls) ^23^, but which did not have regulatory approval. This offered the opportunity to conduct an observational study of drug usage and disease progression from quantitative data recorded by members of the PatientsLikeMe community. The 149 patients who fulfilled inclusion criteria for the study were matched with multiple controls (447 patients in total) based on their prior disease progression. Disease progression was measured using the Revised ALS Functional Rating Scale, which measures patient-reported functional impairment in domains such as speech, swallowing, walking, arm function and respiratory function. No difference in disease progression was observed after 12 months between the overall study group and those patients in the lithium carbonate treatment group (78 patients). Subsequent randomized studies reached the same conclusion that there was no clinical effect in the overall population, although genotype subgroups were associated with variations in response to treatment ^24^. The approach described in this case study has many limitations and cannot be considered a substitute for double-blind RCTs. However, it does suggest that data reported by patients in online health communities may be useful for accelerating clinical discovery and evaluating the effectiveness of drugs already in use.

**Table 3.** Example sources of real world data ^a^.

Data source	Data owners/curators	Typical coverage (patient records)	Typical time to data access
**Administrative claims databases**	HealthCore, Japanese Medical Claims Database, NHS, Optum, Truven,	> 10 million	Immediate
**Electronic health/medical records**	CPRD, Evidera, Flatiron Health, NorthWest eHealth, Optum, Parexel, PCORnet, QuintilesIMS	2–10 million	Immediate
**Clinical registries**	American College of Cardiology, SwedeHeart, CALIBER, CancerLinQ, Health Data Insight, Severe Asthma Registry	< 2 million	Within 1 year
**Prospective studies and hybrid** **approaches**	CROs/AROs, academic partnerships	> 1000	Over 1 year
**Patient-generated data (e.g. social** **media or patient-powered research** **networks)**	PatientsLikeMe, Carenity, PCORnet	> 100 000	Immediate ^b^

^a^Further information on data sources available at: https:///rwe-navigator.eu.

^b^Taking account of privacy and unstructured data considerations.

ARO, academic research organization; CPRD, Clinical Practice Research Datalink; CRO, contract research organization; NHS, National Health Service; PCORnet, National Patient-Centered Clinical Research Network.

A scan for data availability and curation for research projects is a necessary step to ensure that the correct choices are made before designing study concepts. This review step is a well-defined process providing knowledge of vendors or research organizations that are able to provide access to data for research purposes. These data can be procured by the pharmaceutical industry and managed and governed within the industry. Data are gathered into an organization by specific RWE functions, which have been formalized by drawing relevant knowledge, processes and people from more established functions such as epidemiology, health economics and observational research, market access/payer divisions, medical affairs, patient safety and health informatics.

### Data access

Access to real world data can be categorized into three forms: commercial, research collaborations and developmental collaborations. Each form of data access has implications for budget, time and the research objective. In commercial data access, a data asset is already available and may be able to address a research question; a vendor will therefore allow access to the data asset through commercial contracts. If an established commercial process is not defined, owing to local regulations regarding the commercialization of either a data asset or an academic affiliation, research collaborations can facilitate access to the data. This might be the case for access to clinical registry data and to general data in Europe and Asia. Finally, in a developmental collaboration there is a focus on working with a group to develop a data asset that can meet the needs of a research project or the design of a prospective study that will enable the curation of specific data elements from patients in a real world setting.

### Data quality

Real world data must be robust and of high quality to generate valuable evidence that meets the need of healthcare decision-makers. Several guidelines have been developed in recent years to aid investigators in the design and execution of real world studies. Currently, there is still no widely accepted consensus as to which one should be used ^25^. From the perspective of industry sponsors of studies that generate real world evidence, there are many considerations that go beyond scientific, medical or methodological quality in the planning, generation and communication of RWE ( Table 4) ^26– 29^. Ultimately, the pharmaceutical industry must conduct research that provides the data or evidence that is required, that is acceptable to healthcare decision-makers and that leads to optimal health outcomes for patients while avoiding the misuse of resources.

**Table 4.** Considerations in the generation of high-quality real world data.

Stage of the RWE journey	Considerations
**Planning**	• Understand the needs of local healthcare decision-makers • Collaborate with external experts for advice on study designs and access to real world data • Generate a comprehensive RWE study (and publications) plan aligned to company strategy and local evidence needs • Be aware of local limitations: constraints in budget, time, drug exposure; logistics and study delivery; availability/willingness of patients and investigators to participate in the study • Identify a priori the potential sources of bias or confounding factors and identify measures to minimize them • Determine if data are required from a single country or multiple countries • Define research question *a priori* following FINER and PICO criteria ^33^
**Generation**	• Select the most appropriate study design and data source to address the research question, considering the strengths and limitations of each: – primary versus secondary data collection – prospective, retrospective or hybrid approach – randomization – descriptive versus analytic – cohort, case-control or cross-sectional study • Evaluate the benefits, risks and consequences to healthcare decision-makers of the selected study design and data source
	• Clearly define the primary and secondary objectives or endpoints • Evaluate the potential for missing data
	• Train internal and external study teams and investigators • Assess availability of suitable data extraction, management and analytics resources • Follow the FAIR data principles when appropriate (e.g. when machines are used to find and use reusable data) ^26^ • Evaluate site monitoring, source data verification and quality aspects in primary data collection studies
**Communication**	• Report methodology and data sources • Ensure transparency in publications strategy • Commit to publication, regardless of the results • Follow best practice guidelines and recommendations (e.g. STROBE, MOOSE, RECORD) ^27– 29, 34, 35^

FAIR, Findability, Accessibility, Interoperability and Reusability; FINER, Feasible to answer, Interesting, Novel, Ethical, Relevant; MOOSE, Meta-analysis Of Observational Studies in Epidemiology; PICO, Patients, Intervention, Comparators, Outcomes; RECORD, REporting of studies Conducted using Observational Routinely-collected health Data; RWE, real world evidence; STROBE, STrengthening the Reporting of OBservational studies in Epidemiology.

## Communication of RWE

Generating robust, high-quality RWE is not sufficient on its own; the pharmaceutical industry must also use this evidence effectively and within frameworks defined by regulators at a country or regional level. In 2017, the FDA released draft guidance on proactive communication of healthcare economic information from the pharmaceutical industry to payers; this was a response to the revision of Section 114 of the FDA Modernization Act through the 21st Century Cures Act ^30, 31^. Section 114 was written to enable the pharmaceutical industry to communicate healthcare economic information more readily to payers and formulary decision-makers, lowering the threshold required for proactive communication from ‘substantial evidence’ to ‘competent and reliable scientific evidence’. The scope of the legislation does not extend to the proactive communication of clinical comparisons; here, the ‘substantial evidence’ threshold still applies, requiring evidence from RCTs. The proactive use of healthcare economic information permitted through Section 114 does not extend to communication with healthcare professionals or patients. In addition to regulatory limitations, and in contrast to the evidence generated by RCTs, healthcare decision-makers may not be aware of what RWE is or how to interpret it. The pharmaceutical industry may be challenged on the robustness of their RWE, perhaps owing to concerns over a lack of randomization in the study of interest or a perception that bias cannot be addressed in real world studies ^32^. Variations in terminology and a lack of transparency in reporting real world studies add to the challenges in communicating the value of RWE to healthcare decision-makers. Several organizations have established initiatives with the objective of raising awareness of RWE and providing training. The GetReal project, established in Europe by the Innovative Medicines Initiative, brought together representatives from the pharmaceutical industry and other healthcare stakeholders to develop resources and training that provide guidance in the planning, generation and communication of RWE. A recent editorial has highlighted how certain challenges in communicating RWE can be overcome ^36^. There is a need for greater transparency in reporting how evidence from a real world study is generated, such as explaining the choice of data source or methodology applied. These efforts will help to ensure that healthcare decision-makers can make informed decisions when assessing RWE alongside evidence from RCTs.

## Conclusions

RWE complements the evidence generated by RCTs and provides healthcare decision-makers with the confidence to choose the right treatment options for patients. Established types of RWE, such as post-marketing safety surveillance, will continue to evolve, adding value to the evidence base for marketed products, and RWE is now embedded and evolving in the reimbursement and regulatory spaces. Beyond this, however, there is an opportunity for positive disruption in pharmaceutical organizations, where decisions and the execution of clinical development, pipeline prioritization and early development can be driven by RWE. This disruption may reduce barriers for drug development, pushing the pharmaceutical industry to become more agile and innovative as it targets increasingly specific patient populations at an unprecedented pace. While companies recognize the need for RWE, greater strategic direction is needed to maximize its impact on health outcomes and commercial success. The challenge for industry is to adapt in order to utilize the full range of RWE, appropriately, in an environment of changing technology and regulations. Industry also has a responsibility, together with academic support, to make use of its knowledge in ambitions to drive the evolution of medicine development and to disrupt the way evidence is generated. Strategic coordination among local markets, global organizations and external collaborators will raise data quality standards and build international confidence in the planning, generation and communication of RWE.
