# Supplementary material for: Real world evidence (RWE) – a disruptive innovation or the quiet evolution of medical evidence generation?
Source: F1000Res. 2018 Aug 29;7:111. Originally published 2018 Jan 25. [Version 2] doi: 10.12688/f1000research.13585.2 (PMC6039945; doi:10.12688/f1000research.13585.2)
Supplement: Peer review history

# Peer review: Referee response for version 1

**DOI:** 10.5256/f1000research.14756.r35542

**Published:** 2018-07-02

**Authors:** Mattias Kyhlstedt

**Affiliations:** 1 Synergus AB, Stockholm, Sweden

The article provides a good overview of the topic.

 I would suggest the following adjustments / comments:

- Following this statement “Owing to the mechanism of data collection and experimental design, RWE studies generally cannot yield definitive causal inference because of the many confounders of variability.”
  - I think it would be good to reference Eichler et al., “Bridging the Efficacy–Effectiveness Gap.” ^1^. About the implications of moving from traditional RCT to RWE and the implications that comes with this. i.e one should actively consider the confounders, not only identify that is a potential problem.

- Table 1:
  - Being the co-author of the article : “Deriving more value from RWE to ensure timely access of medicines by patients” ^2^. I think this would be an appropriate reference. I would suggest that this would add the following perspective:
    - The structured search to identify the relevant real world data sources as an essential step for successful RWE execution.
- Table 4:
  - In the planning phase it is essential to understand the confounding factors that may impact the outcome question of interest in order to take this in to account in the study design / evaluation of the RWE study.

I have read this submission. I believe that I have an appropriate level of expertise to confirm that it is of an acceptable scientific standard.

## References

[1] EichlerHAbadieEBreckenridgeAFlamionBGustafssonLLeufkensHRowlandMSchneiderCBloechl-DaumB: Bridging the efficacy–effectiveness gap: a regulator's perspective on addressing variability of drug response. *Nature Reviews Drug Discovery*.2011;10(7) : 10.1038/nrd3501 495-506 10.1038/nrd3501 21720406

[2] AnderssonSWKyhlstedtM: Deriving more value from RWE to ensure timely access of medicines by patients. *J Comp Eff Res*.2017;6(5) : 10.2217/cer-2017-0030 391-395 10.2217/cer-2017-0030 28699769

# Peer review: Referee response for version 1

**DOI:** 10.5256/f1000research.14756.r30250

**Published:** 2018-01-29

**Authors:** Marc L. Berger

**Affiliations:** 1 SHYFT Analytics, Waltham, MA, USA

This is a nice summary of the state of play with respect to real world evidence.

 Some minor changes and additions would be worthwhile:

- Page 3:  2nd paragraph, last sentence: Owing to the mechanism of data collection and experimental design, RWE studies generally cannot yield definitive causal inference...." The word cannot should be changed to may not. It is a matter of discussion whether one can impute causality vs causation from observational studies (see Berger et al. ^1^)
- Page 5: Discussion of Phase 1-3 Clinical Study Design and Table 2: RWE can inform clinical study design in other ways including assessing size of recruitment pool of patients with different sets of inclusion or exclusion criteria; estimating treatment effect size for sample size estimation; simulating trials in advance; and recruitment of sites.
- Page 7: Table 3: Next to Electronic health/medical records, Flatiron Health should be added to the list data owner/curators. They are currently working with the FDA on data quality issues.
- Page 8, Table 4: Next to communication, the authors should cite the various ISPOR publications on the subject Including:
  - Caro et al. ^2^;
  - Recommendations from the joint ISPOR‐ISPE Special Task Force on real‐world evidence in health care decision making, co-published in 2 papers in ViH ^3^ and PEpi and Drug Safety ^4^;
  - Wang et al. ^5^^,^^6^.

I have read this submission. I believe that I have an appropriate level of expertise to confirm that it is of an acceptable scientific standard.

## References

[1] BergerMLDreyerNAndersonFTowseASedrakyanANormandSL: Prospective observational studies to assess comparative effectiveness: the ISPOR good research practices task force report. *Value Health*.15(2) : 10.1016/j.jval.2011.12.010 217-30 10.1016/j.jval.2011.12.010 22433752

[2] Jaime CaroJEddyDMKanHKaltzCPatelBEldessoukiRBriggsAHISPOR-AMCP-NPC Modeling CER Task Forces: Questionnaire to assess relevance and credibility of modeling studies for informing health care decision making: an ISPOR-AMCP-NPC Good Practice Task Force report. *Value Health*.2014;17(2) : 10.1016/j.jval.2014.01.003 174-82 10.1016/j.jval.2014.01.003 24636375

[3] BergerMLSoxHWillkeRJBrixnerDLEichlerHGGoettschWMadiganDMakadyASchneeweissSTarriconeRWangSVWatkinsJMullinsCD: Good Practices for Real-World Data Studies of Treatment and/or Comparative Effectiveness: Recommendations from the Joint ISPOR-ISPE Special Task Force on Real-World Evidence in Health Care Decision Making. *Value Health*.2017;20(8) : 10.1016/j.jval.2017.08.3019 1003-1008 10.1016/j.jval.2017.08.3019 28964430

[4] BergerMLSoxHWillkeRJBrixnerDLEichlerHGGoettschWMadiganDMakadyASchneeweissSTarriconeRWangSVWatkinsJDaniel MullinsC: Good practices for real-world data studies of treatment and/or comparative effectiveness: Recommendations from the joint ISPOR-ISPE Special Task Force on real-world evidence in health care decision making. *Pharmacoepidemiol Drug Saf*.2017;26(9) : 10.1002/pds.4297 1033-1039 10.1002/pds.4297 28913966PMC5639372

[5] WangSVSchneeweissSBergerMLBrownJde VriesFDouglasIGagneJJGiniRKlungelOMullinsCDNguyenMDRassenJASmeethLSturkenboomMjoint ISPE-ISPOR Special Task Force on Real World Evidence in Health Care Decision Making: Reporting to Improve Reproducibility and Facilitate Validity Assessment for Healthcare Database Studies V1.0. *Pharmacoepidemiol Drug Saf*.26(9) : 10.1002/pds.4295 1018-1032 10.1002/pds.4295 28913963PMC5639362

[6] WangSVSchneeweissSBergerMLBrownJde VriesFDouglasIGagneJJGiniRKlungelOMullinsCDNguyenMDRassenJASmeethLSturkenboomMjoint ISPE‐ISPOR Special Task Force on Real World Evidence in Health Care Decision Making: Reporting to Improve Reproducibility and Facilitate Validity Assessment for Healthcare Database Studies V1.0. *Value Health*.2017;20(8) : 10.1016/j.jval.2017.08.3018 1009-1022 10.1016/j.jval.2017.08.3018 28964431
